# Investigation of distinct gene expression profile patterns that can improve the classification of intermediate-risk prognosis in AML patients

**DOI:** 10.3389/fgene.2023.1131159

**Published:** 2023-02-14

**Authors:** Nasr Eshibona, Michelle Livesey, Alan Christoffels, Hocine Bendou

**Affiliations:** SAMRC Bioinformatics Unit, South African National Bioinformatics Institute, University of The Western Cape, Cape Town, South Africa

**Keywords:** acute myeloid leukemia, risk classification, intermediate-risk, gene expression, prognosis

## Abstract

**Background:** Acute myeloid leukemia (AML) is a heterogeneous type of blood cancer that generally affects the elderly. AML patients are categorized with favorable-, intermediate-, and adverse-risks based on an individual’s genomic features and chromosomal abnormalities. Despite the risk stratification, the progression and outcome of the disease remain highly variable. To facilitate and improve the risk stratification of AML patients, the study focused on gene expression profiling of AML patients within various risk categories. Therefore, the study aims to establish gene signatures that can predict the prognosis of AML patients and find correlations in gene expression profile patterns that are associated with risk groups.

**Methods:** Microarray data were obtained from Gene Expression Omnibus (GSE6891). The patients were stratified into four subgroups based on risk and overall survival. Limma was applied to screen for differentially expressed genes (DEGs) between short survival (SS) and long survival (LS). DEGs strongly related to general survival were discovered using Cox regression and LASSO analysis. To assess the model’s accuracy, Kaplan-Meier (K-M) and receiver operating characteristic (ROC) were used. A one-way ANOVA was performed to assess for differences in the mean gene expression profiles of the identified prognostic genes between the risk subcategories and survival. GO and KEGG enrichment analyses were performed on DEGs.

**Results:** A total of 87 DEGs were identified between SS and LS groups. The Cox regression model selected nine genes CD109, CPNE3, DDIT4, INPP4B, LSP1, CPNE8, PLXNC1, SLC40A1, and SPINK2 that are associated with AML survival. K-M illustrated that the high expression of the nine-prognostic genes is associated with poor prognosis in AML. ROC further provided high diagnostic efficacy of the prognostic genes. ANOVA also validated the difference in gene expression profiles of the nine genes between the survival groups, and highlighted four prognostic genes to provide novel insight into risk subcategories poor and intermediate-poor, as well as good and intermediate-good that displayed similar expression patterns.

**Conclusion:** Prognostic genes can provide more accurate risk stratification in AML. CD109, CPNE3, DDIT4, and INPP4B provided novel targets for better intermediate-risk stratification. This could enhance treatment strategies for this group, which constitutes the majority of adult AML patients.

## 1 Introduction

Acute myeloid leukemia (AML) is a hematologic cancer characterized by clonal proliferation and the accumulation of immature myeloid progenitors (Arber et al., 2016). AML is the most prevalent leukemia subtype in adults. The disease is highly heterogeneous with a variable prognosis and a high mortality rate (Gregory, 2009; Vakiti and Mewawalla, 2022). Recent intensive research in genomics, novel treatments, and prognostic markers have substantially improved our understanding of many of the biological aspects of this complex disease (Green and Heiko, 2020). However, the global outcome of AML patients remains poor (Wheatley et al., 2009).

The revised European LeukemiaNet (ELN) risk classification system, categorizes newly diagnosed AML patients into favorable-, intermediate-, and adverse-risk groups based on cytogenetic and molecular profiles, which serves as a guideline to establish treatment strategies (Döhner et al., 2022). However, it has been noted that this classification system does not completely reflect the heterogeneity within each subgroup. In particular, the intermediate-risk group exhibits significantly diverse biology and prognosis (Hu et al., 2021).

A poorly defined intermediate-risk group results in the majority of AML patients being stratified to an intermediate-risk category (an umbrella category) because they do not meet the criteria that identify specific entities of established prognostic relevance (Awada et al., 2022). Intermediate-risk AML patients feature heterogeneous clinical outcomes, and it further remains a challenge to assign a suitable consolidation of therapy (Döhner et al., 2015; Hu et al., 2021). This emphasizes the need for a more comprehensive description and understanding of the genetic basis of the intermediate-risk group to improve AML patients’ prognosis and provide more effective treatment strategies.

The original aim of the ELN genetic categories was to standardize reporting of genetic abnormalities, particularly for correlations with clinical characteristics and outcomes. However, significant modifications to the risk classification for AML from 2017 (Döhner et al., 2017) to 2022 revision (Döhner et al., 2022), which excluded the FLT3-ITD mutation, shows that the diagnosis and management of the intermediate-risk group, in particular, remain inexact. Generally, the AML classification and prognostic criteria are based on cytogenetic and molecular features at the time of diagnosis, and thus studies tend to exclude prognostic stratification and base the distinction between the intermediate-I and intermediate-II categories solely on genetic characteristics (Döhner et al., 2010). Meanwhile, a subsequent study demonstrated longer OS in the intermediate-I group than in the intermediate-II group, however, the two groups were prognostically indistinguishable in older patients, who constitute most AML cases (Mrózek et al., 2012).

The purpose of this study is to facilitate improved intermediate-risk stratification of AML and also focus on prognostication. The gene expression profiles of AML patients were investigated to identify gene signatures that differentiate between short- and long-term survival for patients categorized as good- or poor-risk as well as the intermediate-risk group. Therefore, the benefit of this study was twofold i) the study enabled the segregation of intermediate-risk patients into good and poor-prognosis based on distinct gene expression profiles, ii) significant prognostic gene signature was identified to differentiate AML patients with good and poor-prognosis. The identified gene signatures associated with survival in AML patients have the potential to serve as prognostic biomarkers that can aid in the prognosis and monitoring of AML. All contribute to a better understanding of the genetic basis of the disease.

## 2 Materials and methods

### 2.1 Microarray data

**FIGURE 1:**
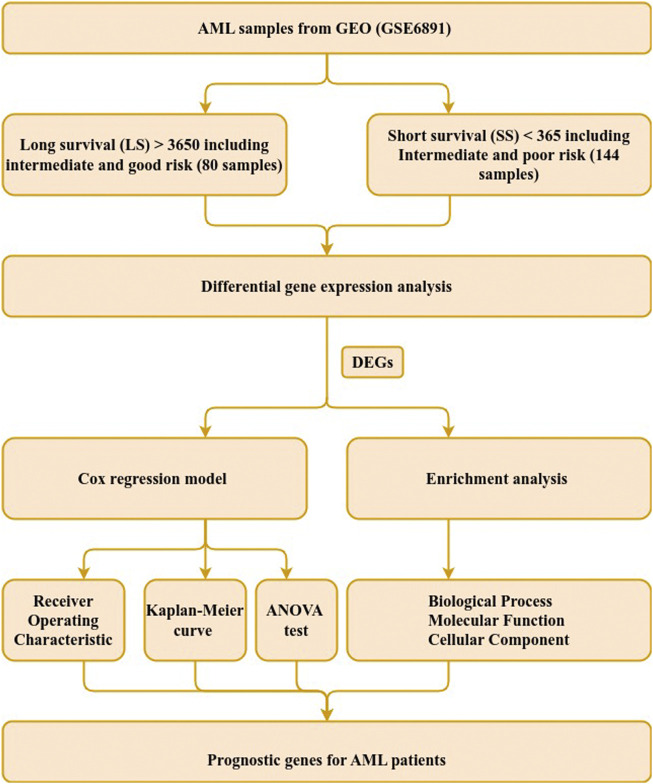
Study workflow. Steps used to identify genetic signature in AML patients with intermediate-risk. The steps comprise data extraction, sample grouping, differential gene expression, survival and functional enrichment analyses. DEGs refer to differentially expressed genes.

The microarray expression profiles of 537 samples and accompanied clinical data were extracted from the Gene Expression Omnibus (GEO) database under the accession number GSE6891 (Verhaak et al., 2009) by the *getGEO* function in the GEOquery R package (version 2.64.2) (Davis and Meltzer, 2007). The patients’ survival data were provisioned by the authors (Verhaak et al., 2009) and samples without clinical data and survival information were excluded from subsequent analyses and 447 samples remained. A complete illustration of the workflow employed in this study is shown in Figure 1.

### 2.2 Samples selection based on risk profile

The patient samples were divided based on OS into short survival (SS) and long survival (LS) (Table 1). The SS includes patients with a survival of less than 365 days, while LS contains patients with a survival of greater than 3,650 days. The two groups were composed by further evaluating the cytogenetic risk classes of the samples, in the clinical file, that were categorized into poor-, intermediate-, and good-risk samples. The SS was further stratified into two risk subcategories: poor (PP) and intermediate-poor (IP) risk, while LS was divided into two risk subcategories: good (GG) and intermediate-good (IG) risk. This additional filtering based on OS and cytogenic risk yielded 224 samples for downstream analysis.

**TABLE 1:** The number of samples stratified by survival time and risk subcategory. Good-Good (GG), Intermediate-Good (IG), Intermediate-Poor (IP), and Poor-Poor (PP) risk of AML sample. Long Survival (LS) and short survival (SS) terms.

Survival time	Risk subcategory	Sample
LS	GG	38
	IG	42
SS	PP	47
	IP	97
Total		224

### 2.3 Data preprocessing

Raw expression data from the 224 selected samples were subjected to background correction, quantile normalization, and log_2_ transformation through the RMA algorithm from the affy R package (version 1.74.0). A filtering operation was applied to reduce the probes that exhibited low variation and a consistently low signal across samples. The median expression of the dataset was calculated and returned a median value of 7.2, thus a probe was kept if the probe expression is above the median in more than 10 samples. The probe identification numbers were then transformed into official gene symbols and duplicate probes were deleted.

### 2.4 Differential gene expression analysis (DGE)

The normalized gene expression of 224 samples and 31,140 genes were analyzed to identify differentially expressed genes (DEGs) between the two survival groups (SS and LS). The limma R package (Ritchie et al., 2015) performs differential gene expression (DGE) analysis and experimental design through linear modeling. The limma package was applied to screen for DEGs that differentiate SS from LS. The DEGs were identified with the parameters of the filter set to | log_2_ fold change |  > 1 and adjusted *p*-value <0.01.

### 2.5 Identification of gene signatures correlated with prognosis

The identified DEGs were subjected to a Cox regression model based on the Lasso algorithm of the glmnet R package (version 4.1-3), to determine which genes were best correlated with patient survival (Friedman et al., 2010; Simon et al., 2011; Tibshirani et al., 2012). The model reduces the number of candidate genes and selected the most significant genes for a patient’s survival, assigning a regression coefficient value to each gene. Genes with a zero coefficient did not affect survival and were discarded. The product of the coefficient value and the corresponding gene’s expression value resulted in a prognostic risk score for each patient in the complete dataset (GSE6891) that provided a survival time. The patient scores were used to calculate a median risk score. A status value of 1 or 0 was assigned to each patient based on whether the patient’s score was greater than or less than the median risk score.

Using Kaplan-Meier (K-M) survival analysis, the prognostic difference between the short- and long-term survival groups was calculated. The K-M curves were created using the *ggsurvplot* function from the survminer R package (version 3.4-0). Additionally, the predicting power (sensitivity and specificity) of the prognostic gene signatures was calculated using the receiver operating characteristic (ROC) curve analysis (Florkowski, 2008). The ROC curves with the observing AUC values were created in Python by applying the metrics.roc_curve function from sklearn using logistic regression algorithms. The results of the Cox regression model were subjected to a validation step using an independent dataset (GSE37642). This test dataset comprises 11 favorable, 78 intermediate, and 35 adverse cytogenetic risk samples. The survival data were inquired and provided by the authors (Herold et al., 2018). K-M curves and Hazard Ratio (HR) of the prognostic genes were generated for the test dataset.

### 2.6 One-way ANOVA

The statistical analysis was performed using the stats R package (version 4.2.1). The statistics were conducted to evaluate for differences in the mean expression profiles of the prognostic genes identified by Cox regression analysis between the survival groups (SS and LS) and risk subcategories. One-way analysis of variance (ANOVA) was applied, followed by Tukey’s *post hoc* test for pairwise comparisons (Tukey, 1949). The null hypothesis (H_0_) of equal mean between the risk subcategories and survival groups was accepted if the *p*-value >0.05; H_0_: there is no significant difference among the group means.

### 2.7 Functional enrichment analyses

List of DEGs were subjected to functional annotations of Gene ontology (GO) (Ashburner et al., 2000) and Kyoto Encyclopedia of Genes and Genomes (KEGG) pathway enrichment analyses, the *EnrichGO* and *EnrichKEGG* functions were used, respectively, in the clusterProfiler R package (version 4.4.4) (Yu et al., 2012). *p*-value <0.05 was determined as a cut-off criterion for significant enrichment.

## 3 Results

### 3.1 Data extraction and DGE analysis

The selected data set was composed of 144 SS and 80 LS based on the criteria of survival time split set out in Section 3.2 (Table 1) as input for DGE analysis. In the DGE, a total of 31,140 genes were screened for DEGs to differentiate between SS and LS. A total of 87 DEGs were identified, where 69 genes were upregulated and 18 genes were downregulated (Supplementary Table S1).

### 3.2 Identification of prognostic genes

By performing univariate Cox regression analysis between the 87 candidate DEGs and patient survival data of (GSE6891), nine prognostic genes were detected and associated with AML patient survival. The prognostic genes were identified using the LASSO algorithm, which assigns non-zero, positive, or negative coefficients. All nine genes had a positive coefficient (Table 2).

**TABLE 2:** Nine prognostic genes with positive coefficient value.

Gene name	Coefficient value
CD109	0.0875676482
CPNE3	0.0755063783
CPNE8	0.0585824601
INPP4B	0.0554178086
SPINK2	0.0544528326
PLXNC1	0.0483530691
LSP1	0.0344057691
DDIT4	0.0216117829
SLC40A1	0.0009147425

**FIGURE 2:**
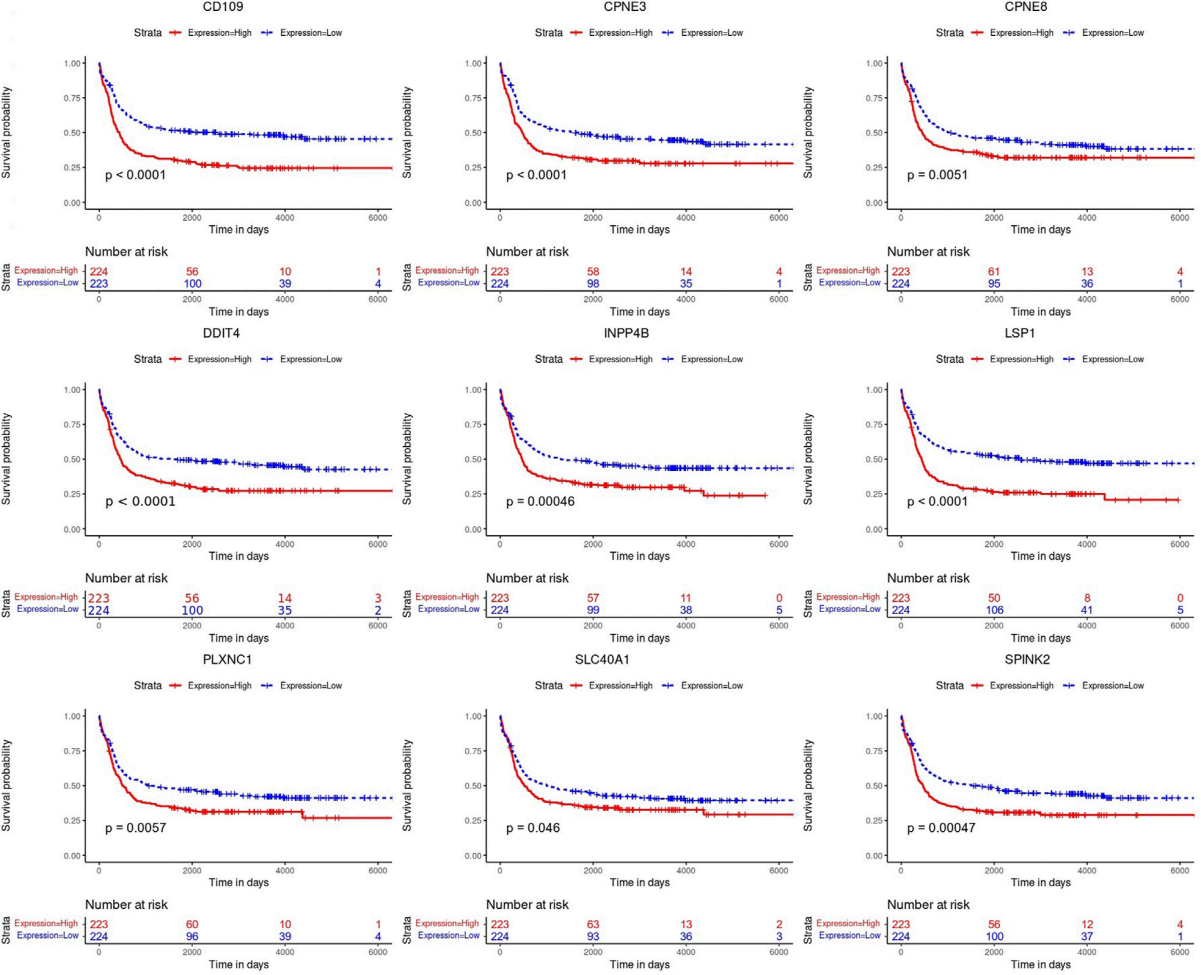
Kaplan-Meier (K-M) survival curves. Analysis revealed the survival prediction associated with high and low gene expression profiles of the prognostic genes in AML patients.

**FIGURE 3:**
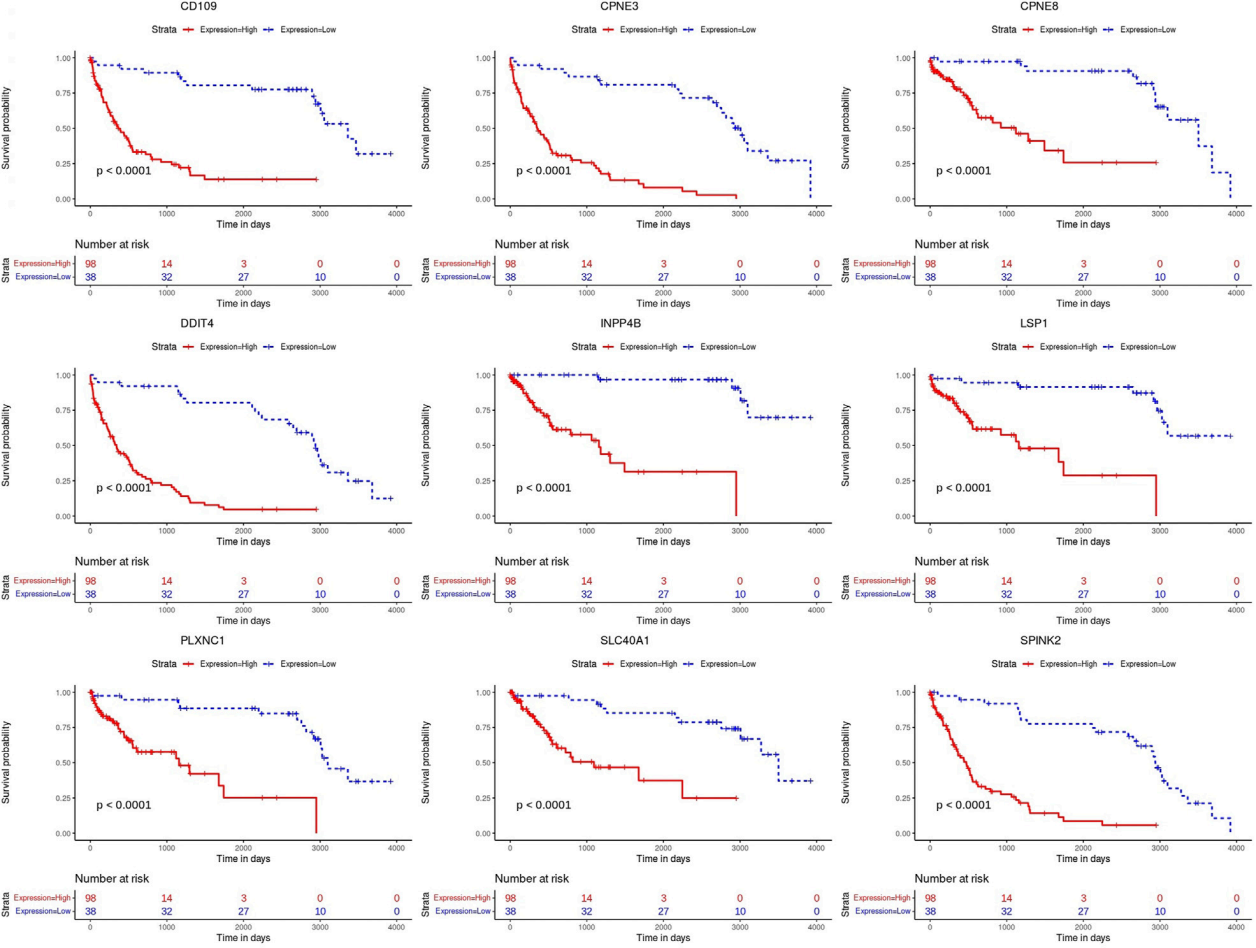
Kaplan-Meier (K-M) survival curves. Analysis on the prognostic genes in the validation dataset (GSE37642).

Kaplan-Meier’s estimates for OS based on patient statuses of each gene with a positive coefficient were derived and presented in Figure 2. All prognostic genes show that a high gene expression level has a poor survival outcome compared to patients with a low gene expression level (Figure 2). The estimates, HR and *p*-value, of the Cox regression model for the prognostic genes were all significant, which confirms the involvement of the alteration in the expression of these genes in the survival of AML patients (Table 3). Additionally, same significant results for K-M and HR were obtained for the validation dataset (GSE37642) (Figure 3; Table 4).

**TABLE 3:** The estimated hazard ratio of each prognostic gene included in the Cox regression for GSE6891 dataset.

Prognostic genes	Hazard ratio	*p*-Value
CD109	0.5322	<0.0001
CPNE3	0.6183	<0.0001
CPNE8	0.7158	0.0051
DDIT4	0.616	<0.0001
INPP4B	0.6572	<0.0001
LSP1	0.5227	<0.0001
PLXNC1	0.7184	0.0062
SLC40A1	0.7875	0.0462
SPINK2	0.6578	0.0005

**TABLE 4:** The estimated hazard ratio of each prognostic gene included in the Cox regression for the independent test dataset (GSE37642).

Gene	HR	*p*-Value
CD109	0.13	<0.0001
CPNE3	0.12	<0.0001
CPNE8	0.13	<0.0001
DDIT4	0.13	<0.0001
INPP4B	0.04	<0.0001
LSP1	0.10	<0.0001
PLXNC1	0.14	<0.0001
SLC40A1	0.17	<0.0001
SPINK2	0.15	<0.0001

### 3.3 Efficiency evaluation of prognostic gene signatures

**FIGURE 4:**
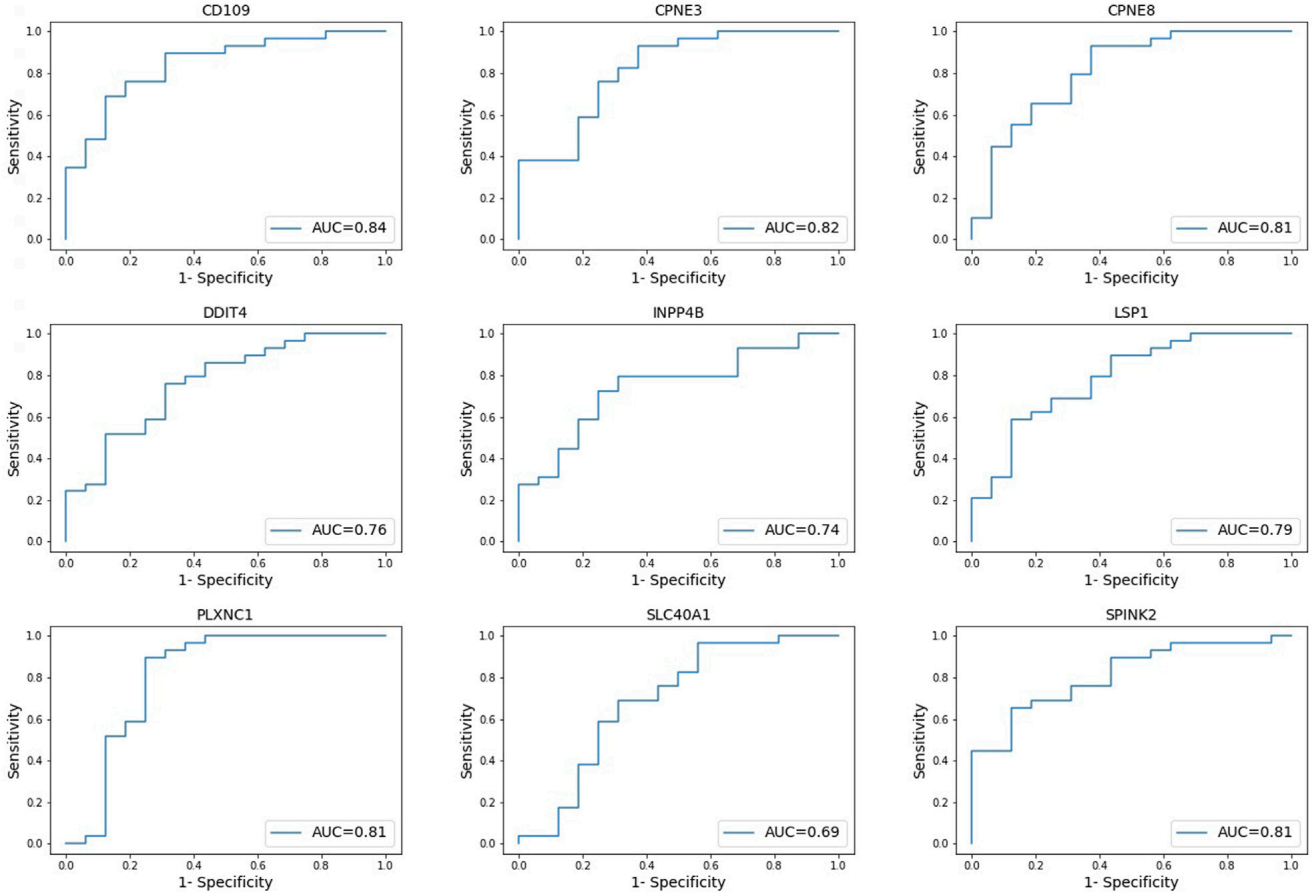
Receiver operating characteristic (ROC) curves. Evaluating the accuracy of high and low gene expression profiles of the nine-genes model in AML patients. *AUC = area under curve.

The prognostic difference between the high and low gene expression profiles of identified prognostic genes in AML patients was also evaluated using ROC curves. ROC analysis evaluated the accuracy of the aforementioned nine-genes model for survival prediction in AML patients. The ROC curve showed the best performance for the area under the curve (AUC) for *CD109* of 0.84. This followed by AUC >0.81 for *CPNE3*, *CPNE8*, *PLXNC1*, and *SPINK2* (Figure 4). Genes *LSP1*, *DDIT4*, and *INPP4B* were 0.74 ≤ AUC ≤0.79, with the lowest AUC of *SLC40A1* was 0.69 (Figure 4).

### 3.4 Gene expression patterns between risk categories

**FIGURE 5:**
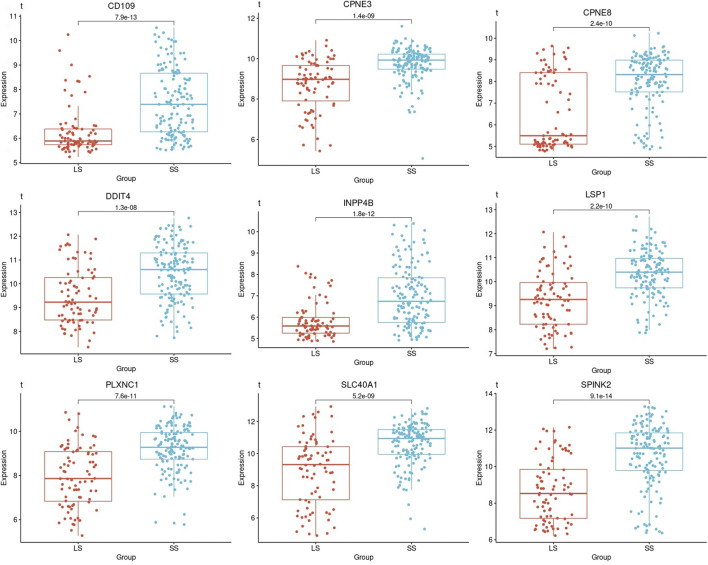
Boxplots based on the survival times of the prognostic genes in AML patients. A boxplot was constructed with the gene expression profile of each prognostic gene in all the samples that were categorized as short- and long-term survival.

One-way ANOVA was used to evaluate for differences in the mean gene expression profiles of each prognostic gene identified between the survival groups and risk subcategories. This includes the difference between the short- (PP and IP) and long-term survival (GG and IG) (Figure 5). ANOVA results confirmed that short- and long-term survival for all prognostic genes are statistically different in gene expression profiles (*p*-value ≤1.3 ${\times 10}^{-8}$ ) (Figure 5).

**FIGURE 6:**
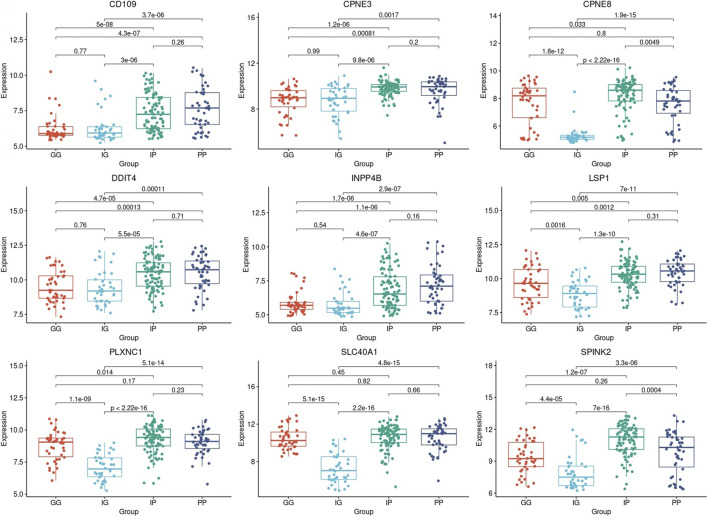
Boxplots based on risk subcategories of the nine prognostic genes in AML patients. A boxplot was constructed with the gene expression profile of each prognostic gene in all the samples that were categorized into the Good-Good (GG), Intermediate-Good (IG), Intermediate-Poor (IP), and Poor-Poor (PP) risk categories.

The samples that were categorized into PP, IP, IG, and GG-risk groups respectively, were investigated for each of the nine-genes models that were identified with prognostic significance. Each risk group was composed of a set of samples in which the gene expression profile of a specific prognostic gene was extracted to construct a boxplot (Figure 6). The differences in the mean gene expression profiles of each prognostic gene identified between PP and IP-risk groups, as well as the GG and IG-risk groups. Also, the difference between the two intermediate-risk groups was evaluated with the IP and IG-risk groups (Figure 6).

All prognostic genes showed a statistically significant difference between the two intermediate-risk groups, i.e., IG and IP-risk (*p*-value ≤5.5 ${\times 10}^{-5}$ ). Also, the ANOVA results between the risk subcategories showed that the mean gene expression profiles of genes *CD109*, *CPNE3*, *DDIT4*, and *INPP4B* showed no statistically significant difference between the PP and IP-risk groups (*p*-value ≥0.16). The same was found for the IG and GG-risk groups (*p*-value ≥0.54) (Figure 6).

### 3.5 Enrichment analysis

**FIGURE 7:**
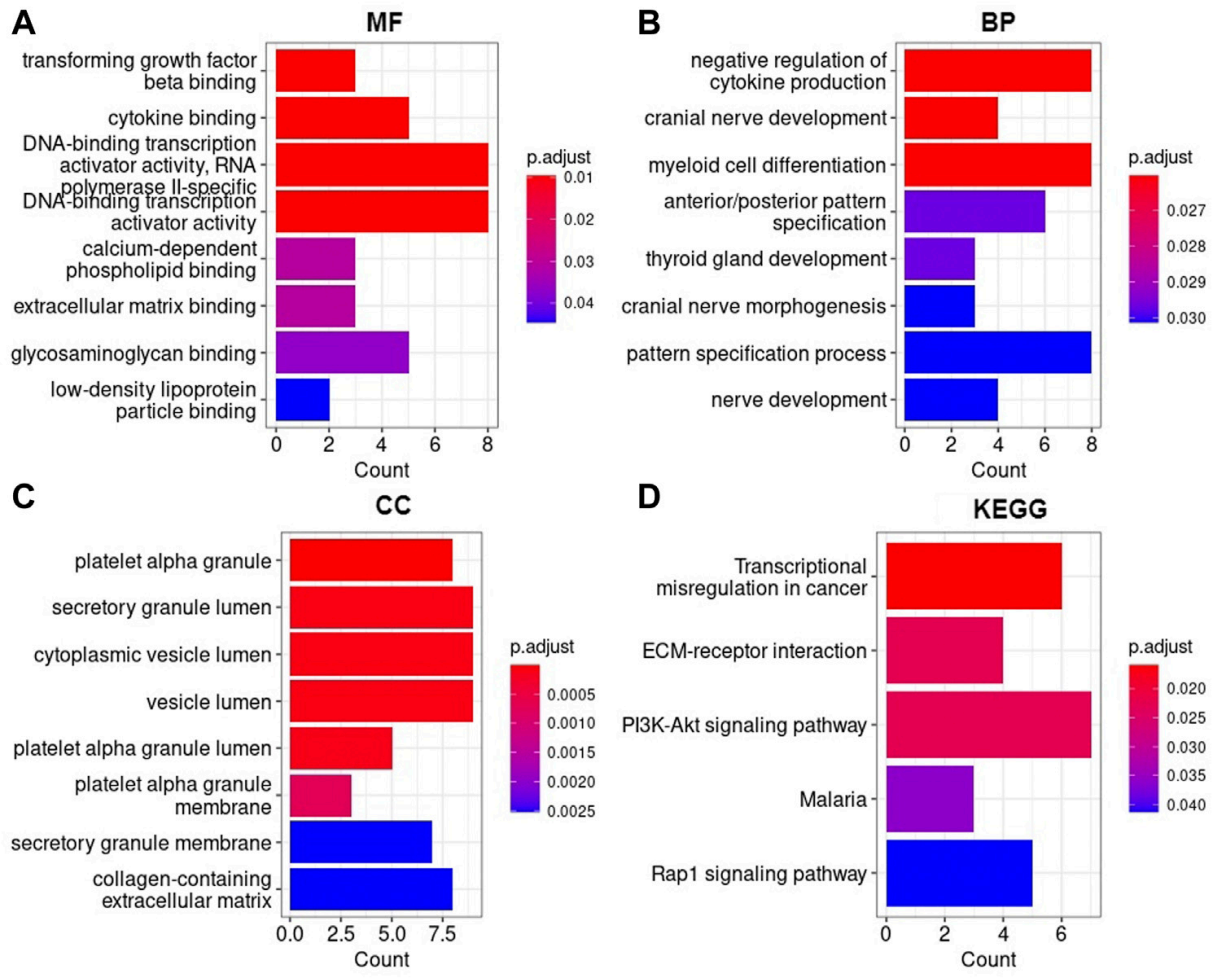
AML DEGs were enriched in Gene Ontology and KEGG pathways **(A)** Molecular function, **(B)** Biological process, **(C)** Cellular component, **(D)** Kyoto Encyclopedia of Genes and Genomes. The horizontal axis represents the number of enriched genes, and the vertical axis represents the gene ontology project and KEGG pathways, respectively.

The GO enrichment analysis showed that AML DEGs were significantly enriched in functional items, such as DNA-binding transcription activator activity, RNA and polymerase II-specific and DNA-binding transcription activator activity, and so on of the biological process (BP). In terms of molecular function (MF), AML DEGs were significantly enriched in functional items such as negative regulation of cytokine production, myeloid cell differentiation, and pattern specification process, among other terms (Figure 7). In terms of the cellular component (CC), AML DEGs were significantly enriched in functional items such as secretory granule lumen, cytoplasmic vesicle lumen, and vesicle lumen (Figure 7). The KEGG analysis indicated significant differences in the transcriptional misregulation in the cancer pathway, PI3K-Akt signaling pathway, and Rap1 signaling pathway (Figure 7).

## 4 Discussion

The risk stratification of AML patients into favorable-, intermediate- and adverse-risk groups is crucial to determine an effective therapy strategy and medical care. However, AML patients continue to feature heterogeneous clinical outcomes, and it remains a challenge to assign a suitable consolidation of therapy. Therefore, it is vital to investigate new leukemogenesis-related characteristics. This study aimed to investigate gene expression profiles in AML patients with long and short survival to decipher the heterogeneity in outcomes of intermediate-risk patients and propose a genetic signature that accurately predict survival of intermediate-risk patients.

The study screened DEGs through the gene expression profiles between short- and long-term survival of AML samples. GO terms and KEGG pathways enrichment analyses was carried out on a total of 87 DEGs to explore the function of the DEGs. GO enrichment analysis illustrated that the DEGs of AML were significantly enriched in functional items such as DNA-binding transcription activator activity, myeloid cell differentiation, secretory granule lumen, cytoplasmic vesicle lumen, and vesicle lumen which was similarly found in studies that focused on predicting disease prognosis for AML (Chen et al., 2021; Chen et al., 2021; Kuang et al., 2021). Interestingly, the prognostic gene *CD109* enriched for all three types of GO terms (BP, MF, and CC). Additionally, the *CD109* gene was enriched in the functional item myeloid cell differentiation, which suggests significant involvement in the development of AML disease.

The KEGG pathway analysis revealed that AML DEGs were enriched in the transcriptional misregulation in cancer, Rap1 signaling pathway, and PI3K-Akt signaling pathway. Consistent with previous studies, the aforementioned pathways have been reported to have an impact on the pathogenesis and prognosis of AML (Martelli et al., 2006; Bertacchini et al., 2015; Yin et al., 2018; Chen et al., 2020). The prognostic *DDIT4* gene enriched in the PI3K-Akt signaling pathway may play a crucial role in the activation of cancer. Therefore, the GO enrichment analysis and KEGG pathway enrichment results showed that the identified DEGs may be important pathogenic genes of AML, contributing to the occurrence and progression of the disease.

This study identified a nine-genes model as potential prognostic biomarkers and therapeutic targets for AML (Table 2). Cox regression and Kaplan-Meier analyses validated the prognostic biomarkers and illustrated that high gene expression of all nine genes has a poor prognosis, whereas a low gene expression is associated with a good prognosis in AML. Therefore, both Kaplan-Meier and high AUC values confirmed that the nine-genes model has good diagnostic efficacy in predicting prognosis for AML. Previous studies supported the findings and reported that the higher expression of the genes is associated with poor prognosis in AML (Woolley, et al., 2015; Fu et al., 2017; Zhao et al., 2018; Gasparetto et al., 2019; Lebedev et al., 2019; Xue et al., 2019; Cheng et al., 2020; Ding et al., 2021). A recent study (Deepak et al., 2022) revealed the potential of *CD109* as a biomarker with diagnostic capabilities in AML, and this study further aligns with this finding, in which CD109 was also found with the highest specificity and sensitivity with AUC (Figure 4).

The difference in mean gene expression profiles of the prognostic genes were evaluated with ANOVA to determine if there is a difference in gene expression profiles between short- and long-term survival samples. ANOVA confirmed a statistically significant difference between the short- and long-term survival in the nine-genes model and therefore confirms the prognostic significance of the nine prognostic genes identified in this study. The intermediate-risk category was further investigated to improve the risk category in which the majority of AML patients are classified. It is noteworthy that all nine prognostic biomarkers displayed a statistically significant difference between the gene expression profiles in the intermediate-good and intermediate-poor risk categories (*p*-value ≤5.5 ${\times 10}^{-5}$ ). The nine prognostic genes are therefore essential in intermediate-risk group classification as AML patients categorized into this risk group could be provided with an improved prognosis.

A crucial finding was made between the gene expression profiles of good-risk compared to intermediate good-risk. It was found that the prognostic biomarkers *CD109*, *CPNE3*, *DDIT4*, and *INPP4B* found in this study displayed the same pattern of gene expression in both GG and IG-risk categories. Hence, GG and IG-risk categories gene expression was not significantly different in the four genes (*p*-value ≥0.54) (Figure 6). The same observation was made when comparing the gene expression profiles of poor-risk and intermediate poor-risk. The same four genes displayed the same pattern of gene expression in both PP and IP-risk categories (*p*-value ≥0.16) (Figure 6). Therefore, the four genes may enable a reclassification of the intermediate-risk category in AML patients into either good- or poor-risk based on the gene expression levels of the four genes. Hence, this finding is important as it could predict the outcome of intermediate risk patients as it is directly associated with survival. This discovery provides a more comprehensive description and understanding of the genetic basis of the intermediate-risk group and therefore has the potential to improve AML patients’ prognosis and provide more effective treatment strategies.

## 5 Conclusion

In this study, we found correlations between risk categories and gene signatures that differentiate short- and long-term survival using gene expression profile data from an AML GEO dataset. The gene expression profiles of nine prognostic genes including *CD109*, *CPNE3*, *DDIT4*, *INPP4B*, *LSP1*, *CPNE8*, *PLXNC1*, *SLC40A1*, and *SPINK2*, showed that high gene expression is associated with poor prognosis. Therefore, the nine genes have the prognostic ability and successfully predict the prognosis of AML patients. Also, the prognostic biomarkers were able to segregate intermediate-risk into poor- and good-risk categories that improve the risk classification by adding prognostic significance to the particular risk category. The prognostic biomarkers *CD109*, *CPNE3*, *DDIT4*, and *INPP4B* provided novel insights as the gene expression pattern were similar between poor and intermediate-poor as well as good and intermediate-good. Therefore, these biomarkers provide targets that can enhance prognosis and provide a more effective treatment strategy for AML patients categorized into the intermediate-risk group. Hence, these biomarkers could serve as potential therapeutic targets in adult AML.

## Data Availability

The datasets presented in this study can be found in online repositories. The names of the repository/repositories and accession number(s) can be found in the article/Supplementary Material.
