# Supplementary material for: Investigation of distinct gene expression profile patterns that can improve the classification of intermediate-risk prognosis in AML patients
Source: Front Genet. 2023 Feb 14;14:1131159. doi: 10.3389/fgene.2023.1131159 (PMC9971493; doi:10.3389/fgene.2023.1131159)
Supplement: Supplementary file 1 [file Table1.docx]

Supplementary Material

Investigation of distinct gene expression profile patterns that can improve the classification of intermediate-risk prognosis in AML patients.

Nasr Eshibona, Michelle Livesey, Alan Christoffels, Hocine Bendou^*^

*** Correspondence: Hocine Bendou** : hocine@sanbi.ac.za

# Supplementary Data

**Supplementary Table 1**: List of significant DEGs between SS and LS. Up-regulation (log_2_  fold change >1 and adjusted *p*-value < 0.01) and down-regulation (log_2_ fold change < -1 and adjusted *p*-value < 0.01)

| PROBEID | SYMBOL | adj.P.Val | logFC |
| --- | --- | --- | --- |
| 203948_s_at | MPO | 6.66433722721692E-07 | -1.72692340999298 |
| 206940_s_at | POU4F1 | 2.99304871047971E-07 | -1.62030038280376 |
| 211341_at | POU4F1 | 4.09282639057784E-09 | -1.50074305365523 |
| 206622_at | TRH | 8.08283334949699E-13 | -1.44338266419843 |
| 203949_at | MPO | 1.95633487577802E-06 | -1.44279703513265 |
| 228827_at | RUNX1T1 | 2.34940598929572E-08 | -1.44068393399573 |
| 205529_s_at | RUNX1T1 | 1.43188160413641E-07 | -1.37944834127139 |
| 210755_at | HGF | 4.61931627940309E-07 | -1.37844488835543 |
| 1556395_at | NA | 1.40712909401244E-08 | -1.27328264275696 |
| 205528_s_at | RUNX1T1 | 4.88149475962196E-09 | -1.22807447972022 |
| 219890_at | CLEC5A | 2.18394017821693E-06 | -1.21314230417013 |
| 209960_at | HGF | 6.37438244373354E-07 | -1.19307588474501 |
| 204885_s_at | MSLN | 1.4285543854801E-06 | -1.09885463573598 |
| 206871_at | ELANE | 0.00289341802260763 | -1.09007669098783 |
| 210997_at | HGF | 2.10185979492379E-07 | -1.08334261066224 |
| 202760_s_at | PALM2AKAP2 | 3.20749222852502E-06 | -1.03662595092478 |
| 206135_at | ST18 | 3.38929160340253E-05 | -1.03656585877682 |
| 226694_at | PALM2AKAP2 | 1.87652495899579E-05 | -1.01188591826101 |
| 209392_at | ENPP2 | 8.01597460509493E-06 | 1.01999968557689 |
| 205608_s_at | ANGPT1 | 3.37073197990753E-05 | 1.02209939714152 |
| 212070_at | ADGRG1 | 2.71179359337343E-09 | 1.02369614629028 |
| 205237_at | FCN1 | 0.000964305606661832 | 1.02891401701537 |
| 228708_at | RAB27B | 1.6645107596387E-06 | 1.03103241488127 |
| 201110_s_at | THBS1 | 0.000912064539401371 | 1.03253023834489 |
| 209555_s_at | CD36 | 0.00142336729432303 | 1.03692773653932 |
| 203523_at | LSP1 | 1.058099465274E-09 | 1.04078578752424 |
| 228766_at | CD36 | 0.00179909758565153 | 1.04481849237915 |
| 206471_s_at | PLXNC1 | 3.47107235490379E-11 | 1.04513420467289 |
| 227856_at | FAM241A | 4.43984044278956E-07 | 1.0486654746159 |
| 205453_at | HOXB2 | 3.12126323737221E-06 | 1.05116104965351 |
| 228372_at | TMEM273 | 2.40792271690105E-06 | 1.05375659972598 |
| 213056_at | FRMD4B | 1.51837648810166E-08 | 1.05925003724532 |
| 202118_s_at | CPNE3 | 6.51205436049427E-11 | 1.06091498007244 |
| 203741_s_at | ADCY7 | 1.73936867121803E-10 | 1.07017594808137 |
| 212386_at | TCF4 | 1.95633487577802E-06 | 1.07400194125269 |
| 211597_s_at | HOPX | 1.99957085724885E-06 | 1.07732077565928 |
| 217853_at | TNS3 | 1.58136813802767E-07 | 1.08247881453307 |
| 205898_at | CX3CR1 | 0.000802424997408272 | 1.08294487642028 |
| 205844_at | VNN1 | 5.81081268921827E-05 | 1.08317054423163 |
| 224596_at | SLC44A1 | 2.20400412506535E-06 | 1.08563302518562 |
| 208792_s_at | CLU | 4.1985776275758E-06 | 1.08637095112643 |
| 205767_at | EREG | 0.00426328570026295 | 1.08937130708731 |
| 217800_s_at | NDFIP1 | 2.07429129794218E-07 | 1.10340104048834 |
| 201669_s_at | MARCKS | 0.000549944485851676 | 1.11140092285802 |
| 235046_at | INPP4B | 1.5632243995606E-07 | 1.11322417808221 |
| 202890_at | MAP7 | 2.08059065739214E-09 | 1.11534179028208 |
| 213110_s_at | COL4A5 | 0.000536846674299879 | 1.11775284448327 |
| 208791_at | CLU | 7.75291783876769E-06 | 1.11997003006859 |
| 225512_at | ZBTB38 | 4.34525531513468E-10 | 1.12388026880831 |
| 210145_at | PLA2G4A | 5.34531176012095E-10 | 1.12875711386747 |
| 206494_s_at | ITGA2B | 3.34364882016399E-08 | 1.14020163802072 |
| 202887_s_at | DDIT4 | 6.25131153334214E-09 | 1.14159491900488 |
| 202119_s_at | CPNE3 | 1.31325682609609E-11 | 1.14602334980558 |
| 1559477_s_at | MEIS1 | 7.0390264882506E-08 | 1.15178101301953 |
| 227236_at | TSPAN2 | 9.18351435683882E-08 | 1.17541076621842 |
| 226545_at | CD109 | 1.427419373503E-08 | 1.2035099909681 |
| 204082_at | PBX3 | 4.21444253796353E-06 | 1.20649096251638 |
| 215646_s_at | VCAN | 0.00770273391925119 | 1.2156232701604 |
| 208029_s_at | LAPTM4B | 2.17321366540848E-06 | 1.22899530426182 |
| 223204_at | GASK1B | 0.000169080411871333 | 1.25167532851227 |
| 212192_at | KCTD12 | 0.000632539040536299 | 1.26913166723962 |
| 238778_at | MPP7 | 1.00181902752795E-09 | 1.27507027869064 |
| 205609_at | ANGPT1 | 8.97389734416107E-06 | 1.28605125089355 |
| 1554679_a_at | LAPTM4B | 1.37248941990576E-06 | 1.2952538339301 |
| 203373_at | SOCS2 | 3.10398572171745E-06 | 1.30015085913583 |
| 205612_at | MMRN1 | 2.14281709311068E-08 | 1.33372158044422 |
| 213241_at | PLXNC1 | 4.3539160907754E-12 | 1.39637739218553 |
| 212314_at | SEL1L3 | 2.00896864576196E-10 | 1.39639885594187 |
| 1553808_a_at | NKX2-3 | 1.39927546742931E-06 | 1.40024148342575 |
| 236738_at | C3orf80 | 1.57316988799411E-07 | 1.40586020285082 |
| 222717_at | CAVIN2 | 6.093405431701E-10 | 1.42325738753958 |
| 203680_at | PRKAR2B | 2.32288150005616E-08 | 1.42460371713351 |
| 203372_s_at | SOCS2 | 2.82759196320902E-06 | 1.44935848946594 |
| 235521_at | HOXA3 | 8.06457710725795E-08 | 1.48780858453495 |
| 217963_s_at | BEX3 | 2.17540302441161E-10 | 1.54509735615073 |
| 204069_at | MEIS1 | 1.77246642396798E-09 | 1.58822863308414 |
| 217975_at | TCEAL9 | 1.15738767302774E-10 | 1.59228413982703 |
| 206478_at | FAM30A | 4.74940257995711E-12 | 1.73105003266287 |
| 213844_at | HOXA5 | 2.36042250744854E-08 | 1.76682399643656 |
| 228365_at | CPNE8 | 1.41224811931834E-13 | 1.77427718942994 |
| 214039_s_at | LAPTM4B | 8.20184976721563E-08 | 1.79418792260646 |
| 228904_at | HOXB3 | 5.28947169890202E-07 | 1.82206709508076 |
| 201427_s_at | SELENOP | 4.54091536457947E-06 | 1.82471579449115 |
| 223044_at | SLC40A1 | 8.08283334949699E-13 | 1.96820903194534 |
| 213150_at | HOXA10 | 8.08283334949699E-13 | 2.02113737641849 |
| 214146_s_at | PPBP | 3.4666938660632E-08 | 2.03848293040479 |
| 206310_at | SPINK2 | 1.41224811931834E-13 | 2.18370800351311 |
